# Investigating the Influence of the Pandemic on the Wandsworth Home Treatment Team

**DOI:** 10.1192/bjo.2022.447

**Published:** 2022-06-20

**Authors:** Ibrahim Ali, Allerdiena Hubbeling, Saurabh Saxena

**Affiliations:** 1 St. George's, University of London, London, United Kingdom; 2 South West London and St. Georges Trust, London, United Kingdom

## Abstract

**Aims:**

This study aimed to determine the impacts of the COVID-19 pandemic on the Wandsworth Home Treatment Team (HTT), South West London and St. George's Mental Health NHS Trust. We hypothesised that demographics and illness characteristics of patients would differ before and during the first wave of the COVID-19 pandemic and that concerns about possible infection with COVID-19 influenced the decision to be referred to the HTT. Additionally, we hypothesised that there would be fewer face-to-face contacts during the initial months of the pandemic.

**Methods:**

Routinely collected data from the trust's electronic records (RiO) were compared from the 15^th^ March – 15^th^ May in both 2019 (control) and 2020 (early pandemic). Patients could have a maximum of 1 variable absent to be included in the study and should have been under the care of the WHTT for longer than 2 days. Overall, 301 patients were included in this study, 181 from 2019 and 122 from 2020. Variables compared were: marital status, age, sex, ethnicity, diagnosis, referral source, referral urgency, referral reason, referral weekday, count seen (number of contacts with a clinician), face-to-face contacts, and length of stay.

**Results:**

The demographic variables: age, sex, marital status, and ethnicity were not significant. Likewise, the length of stay of patients, referral reason, and referral weekday were also not significant. However, during the early pandemic, there was an increase of 11% in the diagnosis of psychotic disorders/psychotic episodes (p = 0.039). Further, the referral urgency of patients within the 2020 period was significantly raised (p=>0.01). The referral source of patients was significantly different with an increased number of patients having been referred to the HTT from the ward (p = 0.017). The mean interactions (count seen) between patients and clinicians significantly lessened from 2019 to 2020, 12.8 Vs 10.2 (p = 0.008). Moreover, the percentage of face-to-face contact had also decreased from 2019 to 2020, 56.1 Vs 46.6 (p = 0.007).

**Conclusion:**

Overall, less patients received care from the home treatment team during the first wave of the pandemic. Age, marital status, sex, ethnicity, length of stay, referral reason, and weekday were not significant. On the contrary, the diagnosis of patients, count seen, face-to-face contacts, referral urgency, and referral source were statistically significant. These findings reflect a different referral pattern to the Wandsworth HTT during the initial months of the pandemic accompanied with fewer face-to-face and other interactions overall.

